# Core–Shell Particle Reinforcements—A New Trend in the Design and Development of Metal Matrix Composites

**DOI:** 10.3390/ma15072629

**Published:** 2022-04-02

**Authors:** Dina V. Dudina, Konstantinos Georgarakis

**Affiliations:** 1 Lavrentyev Institute of Hydrodynamics SB RAS, Lavrentyev Ave. 15, Novosibirsk 630090, Russia; 2 Institute of Solid State Chemistry and Mechanochemistry SB RAS, Kutateladze Str. 18, Novosibirsk 630128, Russia; 3 School of Aerospace, Transport and Manufacturing, Cranfield University, Cranfield MK43 0AL, UK; K.Georgarakis@cranfield.ac.uk

**Keywords:** core–shell particle, reinforcement, metal matrix composite, interface

## Abstract

Metal matrix composites (MMCs) are a constantly developing class of materials. Simultaneously achieving a high strength and a high ductility is a challenging task in the design of MMCs. This article aims to highlight a recent trend: the development of MMCs reinforced with particles of core–shell structure. The core–shell particles can be synthesized in situ upon a partial transformation of metal (alloy) particles introduced into a metal matrix. MMCs containing core–shell particles with cores of different compositions (metallic, intermetallic, glassy alloy, high-entropy alloy, metal-ceramic) are currently studied. For metal core–intermetallic shell particle-reinforced composites, the property gain by the core–shell approach is strengthening achieved without a loss in ductility. The propagation of cracks formed in the brittle intermetallic shell is hindered by both the metal matrix and the metal core, which constitutes a key advantage of the metal core–intermetallic shell particles over monolithic particles of intermetallic compounds for reinforcing purposes. The challenges of making a direct comparison between the core–shell particle-reinforced MMCs and MMCs of other microstructures and future research directions are discussed.

## 1. Introduction

Particles of core–shell structure are attractive as reinforcements owing to a significant difference between the properties of the core and the shell, which can give different functionalities to a composite structure [1]. When a particle is introduced into a matrix, the shell on the particle surface can improve the physical and/or chemical compatibility of the filler with the matrix. Composites with fillers of core–shell structure can be designed to serve a variety of purposes. An example is the development of metal–diamond composites with a high thermal conductivity, in which the diamond fillers are of core–shell structure. The shell on the diamond particles, a carbide coating, is formed to improve the wettability of the filler by the matrix metal [2]. Forming a metallic shell on diamond particles was shown to increase the diamond content in cold-sprayed coatings formed from mixtures of diamond crystals with an aluminum powder [3]. It is also possible to consider an interfacial nanometer-thick layer ensuring bonding between the filler and the matrix and differing in the structure from those as a thin shell [4].

In the present article, we discuss the perspectives of core–shell particles as constituents of metal matrix composites (MMCs). Conventional reinforcements in MMCs are single-phase ceramic or intermetallic particles [5,6,7]. Achieving both a high strength and a high ductility is a persistent challenge in the design of these composites. A trend that has recently emerged in the development of MMCs is to form reinforcements of core–shell structure. In this structure, the core and the shell are different phases (materials) with a distinct interface between them. Core–shell particles can be formed ex situ and then introduced into a matrix. The consolidation of core–shell particles allows forming materials with a structure, in which the layers of the shell constitute a network [8]. However, in the majority of studies, the core–shell structure is a result of a partial in situ chemical reaction of the particles with the matrix. When particles capable of reacting with the matrix metal are relatively large, the core–shell structure will form before the embedded particle is fully transformed.

The core materials can be of different compositions: single metal [9], MXene phase [10], metal-ceramic composite [11], intermetallic phase [12], glassy alloy [13,14,15], high-entropy alloy [16,17] or carbon [18]. Shells forming in situ in the cases of metal (metal-containing) cores are of intermetallic or solid solution nature. The shell can be formed via the interaction of one of the components of the alloy matrix with the core metal [19,20]. 

To the best of our knowledge, a discussion focused on the core–shell particle approach to the strengthening of MMCs was lacking. Without aiming to make a comprehensive list of core–shell particle-reinforced composites manufactured to date, we provide a brief overview of this sub-class of MMCs and analyze the challenges of directly comparing these materials with those of other microstructures.

## 2. Fabrication and Microstructure Formation of Core-Shell Particle-Reinforced Metal Matrix Composites

The in situ formation of core–shell reinforcements has been realized by a number of methods, including solid state sintering of powder mixtures under pressure [11,12,13,14], solid state sintering under pressure followed by hot deformation processing [15] (a combination with pre-heating [16] or post-deformation annealing [9] is possible), sintering of previously obtained metal@metal core–shell particles with an additional amount of the powder of the matrix [21], a solid state reaction between metallic rods and the surrounding matrix [19,20], thixoforming [22,23,24], friction stir processing followed by annealing [25], modification of a pre-existing skeleton followed by infiltration [26], a reaction between solid particles with a metallic melt [27] and a synthesis in a metallic melt from the added reactants [18]. 

**Figure 1:**
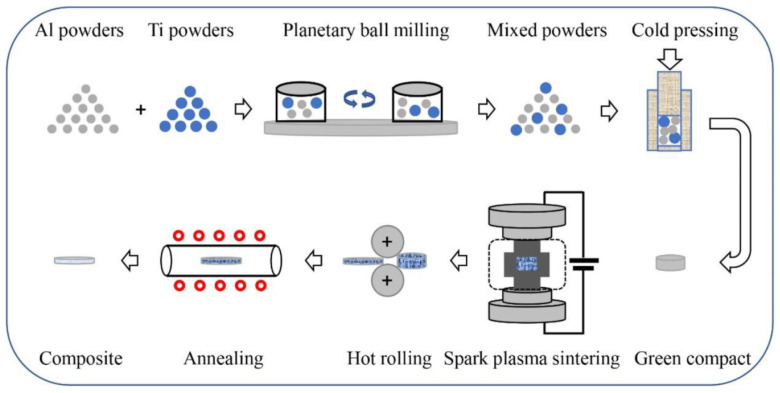
Fabrication route of core–shell particle-reinforced Al matrix composites based on solid state sintering followed by hot rolling and annealing. Reprinted from [9], Copyright (2020), with permission from Elsevier.

**Figure 2:**
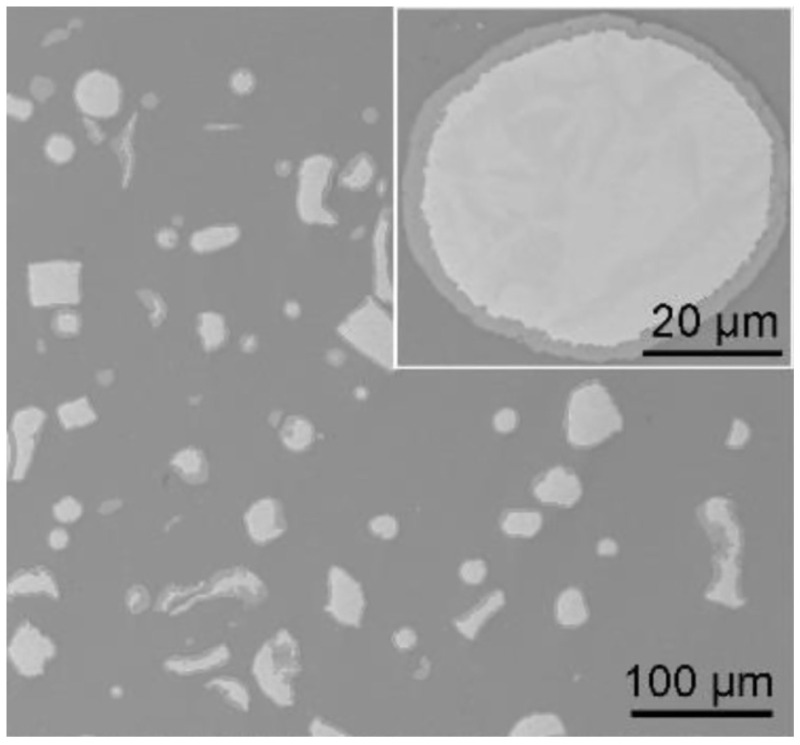
Microstructure of the Ti@Al_3_Ti core–shell particle-reinforced Al matrix composite (annealing of the spark plasma sintered and hot-rolled material at 600 °C for 2 h). Inset: a core–shell particle. Reprinted from [9], Copyright (2020), with permission from Elsevier.

Let us consider the in situ synthesis of composites with core–shell reinforcements using a solid state processing scheme used by Guo et al. [9]. First, Al-Ti composites are obtained by spark plasma sintering (SPS) followed by hot rolling. Then, Ti@Al_3_Ti core–shell particles are formed via a controlled in situ reaction between Al matrix and Ti particles during annealing of the hot-rolled material. The sequence of operations leading to the formation of Ti@Al_3_Ti core–shell particle-reinforced aluminum matrix composites is shown in Figure 1. The microstructure of the composite obtained after 2 h of annealing at 600 °C is presented in Figure 2. The inset demonstrates a particle with a grown shell. The formation of a thin layer of a hard phase at the interface between large particles of the additive and the matrix helps eliminate agglomeration and random clustering of the grains of the hard phase at scales greater than the shell thickness, as the nucleation of grains is strictly determined by the contact surface.

The interaction of two metals appears to be the simplest way to form a hard intermetallic phase. However, this interaction can be prevented by certain factors. When Al+Fe mixtures were sintered at 560 °C, Fe@Al_5_Fe_2_ core–shell particles were the dominant reinforcements in composites sintered under an argon atmosphere, while Fe particles remained as the dominant reinforcements in composites sintered under a nitrogen atmosphere [28]. It was suggested that AlN layers formed on the Al particle surfaces during sintering in nitrogen inhibit diffusion between aluminum and iron.

**Figure 3:**
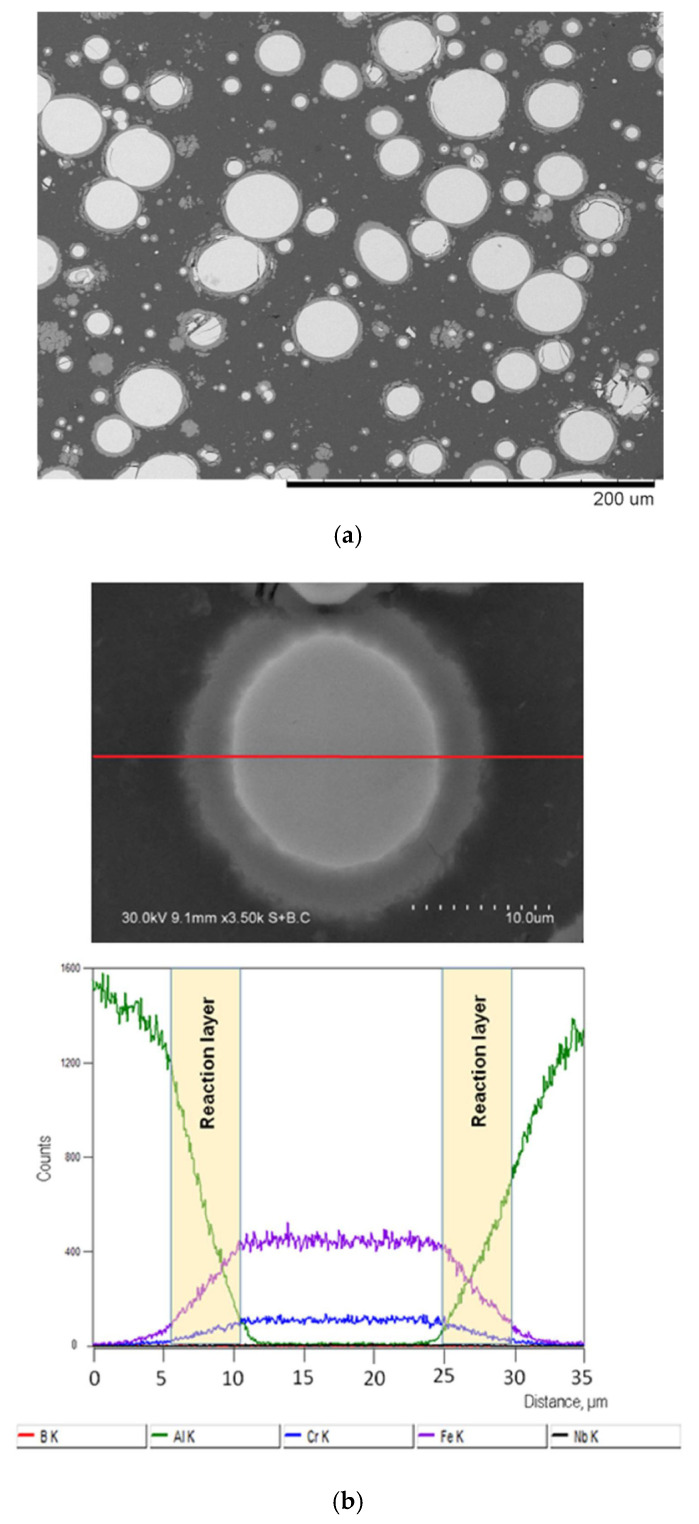
(**a**) Microstructure of the composite obtained by spark plasma sintering of Al-20 vol.% Fe_66_Cr_10_Nb_5_B_19_ powder mixture, 540 °C, 3 min, (**b**) Fe-based alloy particle in an Al matrix, and results of the energy-dispersive spectroscopy analysis along the red line on the image. Reprinted from [14]. This article is an open access article distributed under the terms and conditions of the Creative Commons Attribution (CC BY) license (https://creativecommons.org/licenses/by/4.0/, accessed on 10 March 2022).

Shells forming in situ upon the interaction of the embedded particles with the matrix can be of complex composition when alloys are taken instead of single-phase metals. For example, in our work, after SPS of an Al-20 vol.% Fe_66_Cr_10_Nb_5_B_19_ mixture [14], the shell forming on the Fe-based alloy particles had a gradient structure (Figure 3) and contained both iron and chromium.

A structural design of core–shell particle-reinforced composites is possible such that hard phases are contained in both the shell and the core. Li et al. [11] noted a poor ductility of B_4_C-Al composites caused by a large difference between the elastic moduli of the phases. In order to overcome this problem, a composite reinforcement was suggested, namely, Ti/B_4_C particles. Upon the introduction into an Al alloy matrix and partial interaction with Al, the Ti/B_4_C composite particles acquired a core–shell structure. The Ti core was modified by the introduction of submicron B_4_C particles. Al_3_Ti formed at the interface between the composite core and the Al alloy matrix. Through the formation of Ti/B_4_C composite particles, it was possible to overcome the problem of the poor wettability between Al and B_4_C. Bonding at the interface between Ti and 2024Al matrix was due to an Al_3_Ti shell formed in situ. Micro-cracks forming upon mechanical loading of the composite were confined within the Ti/B_4_C particles.

**Figure 4:**
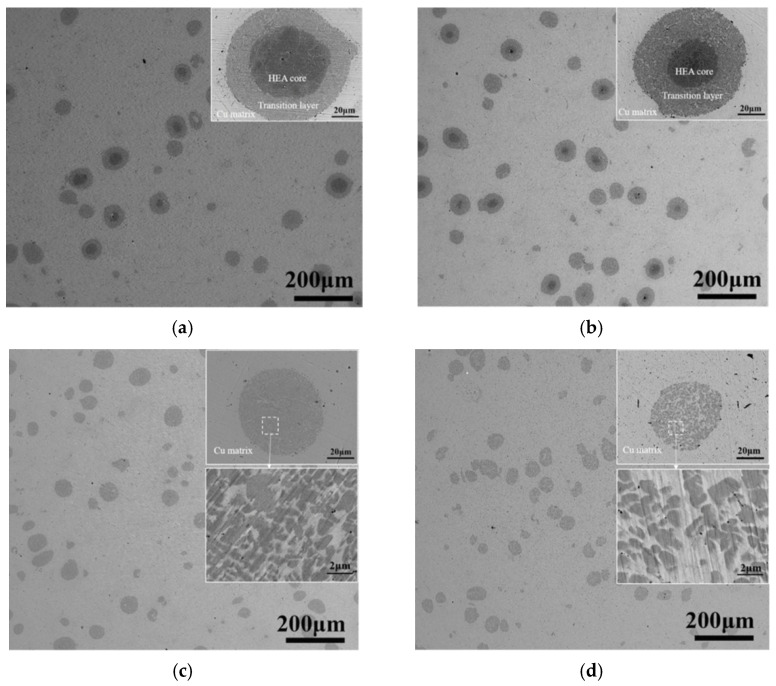
Microstructure of as-extruded composites obtained from a 10 vol.% AlCoCrFeNi–Cu mixture; the sintered composites were preheated for 2 h (**a**), 4 h (**b**), 6 h (**c**) and 8 h (**d**) before extrusion. Insets are the corresponding magnified images of the particles. Reprinted from [16], Copyright (2021), with permission from Elsevier.

Microstructure development scenarios leading not only to the in situ formation of core–shell particles but also to the simultaneous modification of the matrix are possible. When 10 vol.% AlCoCrFeNi–Cu composites were annealed for different periods of time, particles of core–shell structure formed first, then the core–shell structure disappeared, as seen in Figure 4 [16]. The size of particulate inclusions decreased with the annealing time, which was a result of diffusion flows between the alloy and the matrix. In the particles of core-structure, copper was found in the shell, its concentration increasing with increasing treatment time. The concentration of aluminum in the shell decreased with the treatment time. Due to diffusion between the matrix and the embedded particles, the composition of the matrix changed, in contrast to situations when the matrix and the inclusion react to form a layer at the interface only (Ni+Al, Fe+Al, Ti+Al systems). As the matrix was modified with the soluble elements, solid solution strengthening contributed to an enhanced strength of the composite. As the treatment time increased, the hardness of the matrix increased while that of the reinforcing particle decreased. Unlike shells of intermetallic nature, the shells described in ref. [16] were of a face-centered cubic (fcc) structure possessing a high ductility. The formation of a thicker shell led to the formation of a more ductile composite. 

While the core–shell structure of the reinforcing particles forms upon heat treatment, other structural changes can occur as well, influencing the overall behavior of the composites. Therefore, it may be difficult to separate the influence of the core–shell morphology on the mechanical behavior of the composites. Liu et al. [29] obtained in situ core–shell structures by SPS of AlCoCrFeNi–Al powder mixtures. When the sintering temperature was high enough for diffusion, shells formed around the particles. The shell consisted of fcc and body-centered cubic (bcc) phases (not intermetallic phases), which was beneficial for the plasticity of the composites. The in situ formed core–shell structure significantly improved the strength and plasticity of the composite relative to those containing shell-free particles. It was reported that the yield strength of the composite of core–shell structure (sintered from a 5 vol.% AlCoCrFeNi–Al mixture) was higher than that of the composite without a core–shell structure by 43%. It is important to note that the composite without a core–shell structure was sintered at a temperature (540 °C) lower than the temperature needed for the core–shell structure to form (600 °C). Sintering at a higher temperature promoted better consolidation of the matrix itself. During the compression test, the fracture did not occur in the composite with a core–shell structure, while the composite without a core–shell structure fractured at a strain of 36%. Strengthening by matrix segmentation was found to be dominant in a core–shell particle-reinforced composite formed from a mixture containing 20 vol.% AlCoCrFeNi.

## 3. Mechanical Properties of Metal Matrix Composites with Core-Shell Reinforcements

The features of the mechanical behavior of composites reinforced with core–shell particles of different compositions are discussed below. In Table 1 and Table 2, the microstructural features and mechanical properties of selected composites reinforced with core–shell particles are given (in compression and tension, respectively). Ma et al. [12] considered it essential to find a strategy to overcome the strength–ductility trade-off of intermetallic-reinforced MMCs and achieve a high strength without significantly deteriorating ductility. In their study, the reinforcements were composed of a hard core of Ni_3_Al and a double shell of Al_3_Ni and NiAl. The core to the double shell phase sequence was Ni_3_Al-NiAl-Al_3_Ni. When the applied force reached a certain level, plastic deformation occurred in the matrix, which led to a stress concentration at the interface between the matrix and the core–shell structure. Cracks propagated along this interface forcing the core–shell structure to “peel off” the matrix. It was suggested that the core–shell structure reduces local stresses by a wavy path of crack propagation; cracks can initiate and propagate within the core–shell particles causing the fracture of the core–shell structure as a whole. 

According to Guo et al. [9], load transfer strengthening is the dominant mechanism of strength enhancement in Ti@Al_3_Ti core–shell particle-reinforced aluminum matrix composites. An advantage of Ti@Al_3_Ti core–shell particles over Al_3_Ti particles is that both the soft Al matrix and the Ti core hinder the propagation of cracks nucleated in the Al_3_Ti shell, which delays the fracture of the composite. Chen et al. [23] noted that, if the reinforcing particles have a core–shell structure with a soft Ti core and a hard Al_3_Ti intermetallic shell, they should have a strengthening effect similar to that of monolithic Al_3_Ti particles. However, cracks generated in the Al_3_Ti phase upon loading are constrained within the shell, which makes the cracks shorter than those crossing the monolithic Al_3_Ti particles with the same diameter as the core–shell particles. The tips of each crack are blunted in the soft Ti core and Al matrix, and the propagation of cracks is thus delayed.

The Ti@(Al-Ti-Si)–A356 alloy matrix composite prepared by powder thixoforming showed an excellent combination of tensile ductility and strength [22]. An Al-Si-Ti intermetallic shell formed around the Ti cores. These core–shell particles exerted a strengthening effect comparable to that offered by the monolithic (Al, Si)_3_Ti particles, but the ductility was higher in the case of the core–shell reinforcement (Table 2). The Ti core inhibited or delayed crack propagation by blunting the crack tips. It should be noted that, in the study described in ref. [22], the fully reacted particles were of different size and had a distribution character (in the matrix) different from that of the core–shell particles; the reinforcements changed not only in morphology but also in the phase constituents as the processing time increased.

For soft core–hard shell reinforcing particles, Chen et al. [22] suggested calculating the equivalent volume fraction of the reinforcement, $V_{p}^{*}$, by introducing a correction factor, C:

(1) $$V_{p}^{*}=V_{p}\cdot C$$

(2) $$C=\frac{A_{1}E_{1}+A_{2}E_{2}}{E_{1}}$$

where $V_{p}$ is the volume fraction of the reinforcing particles; $E_{1}$ and $E_{2}$ are the elastic moduli of the shell and the core, respectively; and $A_{1}$ and $A_{2}$ are the volume fractions of the shell and the core in the core–shell structure, respectively. According to the modified shear lag theory [30], an increase in the yield strength of a composite reinforced with spherical particles caused by load transfer is determined as:

(3) $$\Delta \sigma_{cy}=\frac{1}{2}V_{p}\sigma_{my}$$

where $\sigma_{my}$ is the yield strength of the unreinforced matrix. In the case of core–shell particles, $V_{p}^{*}$ will be used instead of $V_{p}$:

(4) $$\Delta \sigma_{cy\;core-shell}=\frac{1}{2}V_{p}^{*}\sigma_{my}$$

From (2), it follows that, if $E_{1}\gg E_{2}$, the contribution of the core to the reinforcement volume is very small such that $C\approx A_{1}$ (the reinforcing effect comes mainly from the shell). When the difference between $E_{1}$ and $E_{2}$ is not very large, the core contributes to the reinforcement volume while hindering the propagation of cracks nucleated in the shell.

Ma et al. [31] conducted the microstructure-based numerical simulations of the mechanical behavior of A356 alloy matrix composites reinforced with Ti@Al_3_Ti core–shell particles. A series of two-dimensional representative volume element models were generated by embedding Ti@Al_3_Ti in an A356 matrix. A monolithic Al_3_Ti particle-reinforced A356 matrix composite was also simulated. An appreciable ductility of the Ti@Al_3_Ti core–shell particle-reinforced composite was explained by the simulation results and was due to the uniform distribution of the ductile globular reinforcing particles, which reduced local stresses both on and inside the core–shell particles. As shown in ref. [32], the stress distribution in the micro-region close to the shell is determined by the difference between the elastic moduli of the shell and core materials. The nanostructuring of an intermetallic shell in composites reinforced with ductile core–brittle shell inclusions also slows down the crack development in the shell [33].

**Table 1:** Microstructural features and mechanical properties in compression of selected composites reinforced with core–shell particles.

Starting Powder Mixtures/Components	Description of the Microstructural Features of the Composite	Compressive Yield Strength, MPa	Ultimate Compressive Strength, MPa	Strain at Fracture, %	Ref.	Comments
Al–20 vol.% Ni_3_Al	Al matrix–Ni_3_Al@NiAl-Al_3_Ni core–shell particles	-	213	3	[12]	improved strength
Al–20 vol.% Fe	Al matrix–Fe@Fe_2_Al_5_ core–shell particles	227	273	12.1	[34]	improved strength, medium plasticity
Al–20 vol.% Fe	Al matrix–Fe@Fe_2_Al_5_ core–shell particles (extruded)	373	461	6.1	[34]	improved strength
Al–20 vol.% Al@Cu core–shell particles	Al matrix–Al@ (Al_2_Cu-Al_4_Cu_9_) core–shell particles	285	400	8	[21]	improved strength, medium plasticity
Al–20 vol.% Fe_66_Cr_10_Nb_5_B_19_	Al matrix–Fe-based alloy@Fe_2_Al_5_-Al_3_Fe core–shell particles	-	780	2	[14]	high strength
Al–10 vol.% CoCrFeNi	Al matrix–CoCrFeNi@AlCoCrFeNi core–shell particles	247	265	12.5	[17]	improved strength, medium plasticity
Nb rods–gray cast iron	Gray cast iron matrix–Nb@NbC core–shell rod	1794	2190	11.6	[20]	high strength, medium plasticity

**Table 2:** Microstructural features and mechanical properties in tension of selected composites reinforced with core–shell particles. Data for composites with monolithic particles are given for comparison (in italics).

Starting Powder Mixtures	Description of the Microstructural Features of the Composite	Tensile Yield Strength, MPa	Ultimate Tensile Strength, MPa	Elongation, %	Ref.	Comments
2024Al alloy–10 wt. % Ti/B_4_C	2024Al alloy matrix–(Ti/B_4_C)@Al_3_Ti core–shell particles	214	300	6.3	[11]	improved strength
A356 alloy–Al–Ti	A356 alloy matrix–Ti@(Al-Ti-Si) core–shell particles	268	373	8.3	[22]	improved strength, medium ductility
*A356 alloy*–*Al*–*Ti*	*A356 alloy matrix*–*monolithic (Al, Si)_3_Ti particles; the particles experienced dispersion*	*278*	*380*	*3.1*	[22]	*improved strength*
A356 alloy–Ti	A356 alloy matrix–Ti@(Al-Ti-Si) core–shell particles (after solution treatment)	143	268	17	[24]	improved strength, high ductility
Al–10 vol.% Ti	Al–Ti@TiAl_3_ core–shell particles	156	172	8	[35]	improved strength, medium ductility
Al–10 vol.% Ti	Al–Ti@TiAl_3_ core–shell particles	198	241	19.8	[9]	improved strength, high ductility
Cu–10 vol.% AlCoCrFeNi	Cu-based matrix–AlCoCrFeNiCu core–shell particles (thin shell)	212	270	11.2	[16]	improved strength, medium ductility
Cu–10 vol.% AlCoCrFeNi	Cu-based matrix–AlCoCrFeNiCu core–shell particles (thick shell)	220	280	14.2	[16]	improved strength, high ductility
*Cu*–*10 vol.% AlCoCrFeNi*	*Cu-based matrix*–*CoCrFeNiCu particles*	*265*	*333*	*15.3*	[16]	*improved strength, high ductility*

**Figure 5:**
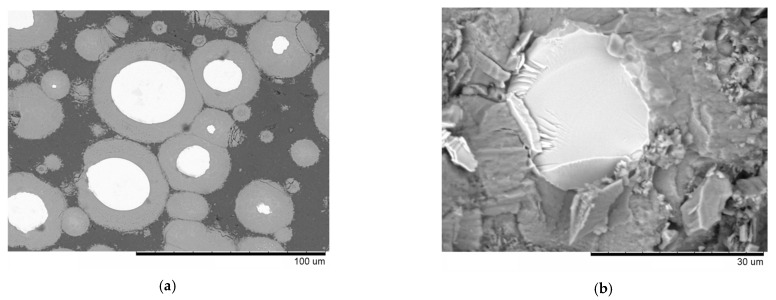
(**a**) Microstructure of the composite obtained by spark plasma sintering of an Al–20 vol.% Fe_66_Cr_10_Nb_5_B_19_ glassy alloy powder mixture, 570 °C, 3 min; (**b**) fracture surface of the composite after a compression test. Reprinted from [14]. This article is an open access article distributed under the terms and conditions of the Creative Commons Attribution (CC BY) license (https://creativecommons.org/licenses/by/4.0/, accessed on 10 March 2022).

As seen in Table 1, in some composites, an increased strength was achieved while retaining an appreciable strain at fracture under compression (aluminum matrix composites [17,21,34] and gray cast iron matrix composites [20]). In ref. [14], a high compressive strength of an Al-based composite was achieved by forming a thick shell, which, at the same time, deteriorated plasticity. Figure 5a shows the microstructure of the composite obtained by SPS of an Al–20 vol.% Fe_66_Cr_10_Nb_5_B_19_ glassy alloy powder mixture [14]. The products of the interaction between aluminum and the metallic glass are Al-Fe-Cr intermetallics. The layer of these products acts as a reinforcing element via the load transfer mechanism, contributing to a greatly increased strength of the composite. The fracture surface of this composite (Figure 5b) shows fractured Fe-based alloy particles (bright areas) and reaction product layers (light-gray areas); both demonstrated a brittle mode of fracture. The matrix showed dimple fracture, which is characteristic of ductile metals. The core–shell particles did not detach from the Al matrix upon fracture of the composite, which indicates the load transfer mechanism in operation. No debonding at the core/shell interface was observed either.

An attractive set of mechanical properties in tension was achieved in some Al-based matrix composites [9,22,24,35] and Cu-based matrix composites [16] (Table 2). A tensile strength of 241 MPa and an elongation of 19.8% were achieved in the composite reinforced with Ti@Al_3_Ti core–shell particles [9]. It is worthwhile to compare the obtained properties with those of a commercially available Al-based alloy with at least one characteristic close to that of the developed composite, in order to assess the advancement made. For that, we can use 201AB-T4 alloy, which is a commercial press-and-sinter Al-based alloy with an ultimate strength of 262 MPa and an elongation of 5% [36]. So, the composite obtained in ref. [9] is much better than 201AB-T4 in terms of ductility and comparable to 201AB-T4 in terms of tensile strength. 

Interestingly, in some cases, a transformation from the core–shell-structured composites to composites without a core–shell structure can lead to better mechanical properties. A transformation of a 10 vol.% AlCoCrFeNi–Cu composite into a CoCrFeNiCu–Cu-based matrix composite was beneficial for both strength and ductility [16] (Table 2). This effect was due to complex nature of the chemical interaction between the embedded particles and the matrix modifying the structure and properties of the matrix.

## 4. Core–Shell versus Alternative Microstructures: The Problem of Comparison

The structural information related to the core–shell particle-reinforced MMCs can be obtained using X-ray diffraction, optical microscopy, scanning electron microscopy, electron back-scattered diffraction and transmission electron microscopy (TEM). The use of TEM helps shed light on the fine structure of the shells, including those consisting of several layers [15,33]. However, when a core–shell particle-reinforced MMC is characterized as a whole or compared with materials of alternative microstructures, certain problems arise. 

In most cases, the core–shell particles embedded in a matrix are not mono-sized and are characterized by a certain size distribution. Therefore, in many studies the exact concentration of the shell material formed in situ is not known, which makes it difficult to calculate the theoretical density of the synthesized composite material.

**Figure 6:**
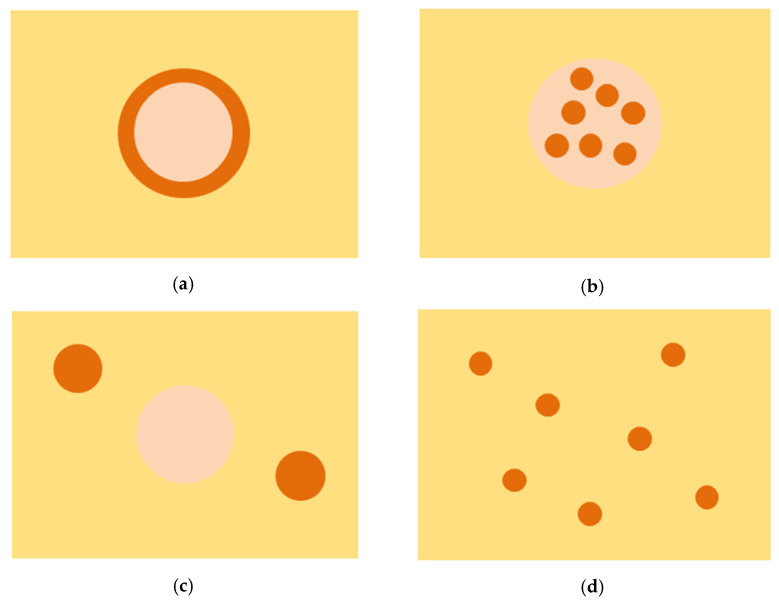
Possible microstructures of metal matrix composites: (**a**–**c**) two materials are embedded in a matrix; the cases differ in the shape of the particles and mutual distribution of the phases, (**d**) a case with particles of the shell material distributed in the matrix (no core material is present). In (**a**), a core–shell particle-reinforced composite is shown. The volume content of the core material in the composite is the same for (**a**–**c**) cases. The volume content of the shell material is the same for (**a**–**d**) cases.

As noted in Section 2, as the core–shell structure forms, other structural changes can occur in the composite system. This makes it challenging to experimentally obtain composites with the same sintering level of the matrix, the same volume content of the reinforcements, the same size and shape of the reinforcements but differing in the structure of the embedded particles. Ideally, in one case, the particles can be of core–shell structure (Figure 6a) with a distinct interface between the core and the shell. In another, they can be composite particles with the same concentrations of the core and shell phases and a structure of intermixed grains with hard particles embedded in a soft material (Figure 6b). Another possibility is to create a composite system with separate particles of the core and shell materials distributed in the matrix (Figure 6c). In this case, the core and the shell materials do not contact each other and cannot perform functions that they perform in the core–shell structures. In Figure 6d, a situation is shown where the shell material reinforcement is used (instead of core–shell particles) in the same volume content as that present in the corresponding core–shell particle-reinforced composite, i.e., no core material is contained in the microstructure. Other geometrical combinations are possible for the same concentrations of constituents. A comparative analysis of the properties of these composites could allow determining the true influence of the reinforcement structure and geometry on the mechanical behavior of the composites and the advantages of the core–shell structure over other possible microstructures.

## 5. Summary and Future Research Directions

MMCs reinforced with particles of core–shell structure are currently presenting a rapidly developing trend. Several research teams have made significant contributions to the understanding of the synthesis mechanisms and the mechanical behavior of these composites. In the present article, we have highlighted the features of the core–shell particle-reinforced composites using examples from the literature and our own research. The analysis of 2015-2022 publications shows that, while it may still be challenging to compare several alternative microstructures and select the “best” structural pattern, the core–shell particle-reinforced MMCs present a promising option owing to the relative ease of fabrication and a good combination of strength and ductility. A rapidly growing number of publications dealing with composites reinforced with core–shell particles indicate a scientific interest in this sub-class of MMCs. 

In our opinion, the following goals should be pursued in forthcoming research:

1. The understanding of the effect of the size and morphology of the particles introduced into a matrix (the volume content of the shell material formed in situ will be higher for smaller added particles). While it may be more convenient to form a reinforcement phase as a shell (layer) on the surface of a large particle (for simplifying the microstructural characterization), the size of the core–shell reinforcements needs to be optimized for practical purposes.
2. The evaluation of the possibilities of intermetallic shell modification to reduce its brittleness (control of grain size and thickness of the shell material, formation of multiple phase shells). The fabrication and investigation of composites with core–shell particles that have strong and ductile shells (composed of metal-based solid solutions of variable composition).
3. The development of simulation approaches to predict and comparatively analyze the mechanical behavior of composites with different microstructures, as the latter may be difficult to do experimentally.
4. The investigation of the features of the formation of core–shell structures during sintering assisted by an electric field, considering the occurrence of local (inter-particle) effects pertaining to those processes. The roughness of the particles is an important parameter, which can be altered by preliminary surface modification of the particles.
5. The evaluation of the technical and economical benefits of the powder metallurgy production of composites with the level of properties offered by the core–shell reinforcements relative to other microstructures and/or technological options.
6. The utilization of MMCs as a medium for the formation of core–shell inclusions, which can be further separated from the matrix by removing (for example, dissolving) the matrix metal. Particles obtained in this manner can be used for other applications (introduced into other matrices or applied as powders of functional materials with core–shell structure).
